# Maternal and fetal outcomes in women undergoing induction of labor with low dose vaginal misoprostol

**DOI:** 10.12669/pjms.39.5.7072

**Published:** 2023

**Authors:** Tanveer Shafqat, Laila Zeb, Syeda Sitwat Fatima, Rehana Bhittani

**Affiliations:** 1 Tanveer Shafqat, FCPS (Obstet & Gynae) Associate Professor, Department of Obstetrics and Gynaecology, Medical Teaching Institute, Lady Reading Hospital, Peshawar, Pakistan; 2 Laila Zeb, FCPS (Obstet & Gynae) Assistant Professor, Department of Obstetrics and Gynaecology, Medical Teaching Institute, Lady Reading Hospital, Peshawar, Pakistan; 3 Syeda Sitwat Fatima, FCPS (Obstet & Gynae) Assistant Professor, Department of Obstetrics and Gynaecology, Medical Teaching Institute, Lady Reading Hospital, Peshawar, Pakistan; 4 Rehana Bhittani, FCPS (Obstet & Gynae) Registrar, Department of Obstetrics and Gynaecology, Medical Teaching Institute, Lady Reading Hospital, Peshawar, Pakistan

**Keywords:** Apgar score, Bishop Score, Eclampsia, Induction of Labor (IOL), Misoprostol

## Abstract

**Objective:**

To determine Maternal and Fetal outcome in women undergoing induction of labour with low dose misoprostol.

**Method:**

A cross-sectional study was carried out to determine the efficacy of Misoprostol for induction of labor (IOL) in MTI, Lady Reading Hospital (LRH), Peshawar from 21st January to 31st December 2021. All pregnant women with singleton pregnancy and cephalic presentation admitted for Induction of Labor were included in the study. Maternal and Fetal outcome was noted. Induction of labor was started with 25 micrograms of Misoprostol, repeated every six hours depending on Bishop Score.

**Results:**

Three hundred and thirty-seven women were included in this study. The majority of females (76%) were in 18-35 years age group. In 92.3% of females, the Bishop score was less than six. The maximum number of females (33.5%) delivered after eight hours of IOL. Sixty-six (66.46%) of females had gestational age of 37-40 weeks. Premature rupture of membranes was the most common indication (32.9%). Three doses of misoprostol were required in 31.2% of females. Only 5.6% of females required six doses of misoprostol for induction. With Misoprostol 85.1% of females delivered spontaneously, 2.37% required forceps delivery, 1.7% required vacuum delivery, and 10.68% delivered by Caesarean Section. APGAR score was 8 /10 in 84% of neonates at birth. Eighty-seven %(87.8%) of neonates did not require NICU admission.

**Conclusion:**

Misoprostol is a safe medicine to be used to induce labor in females. It can help shorten the duration of labor, with good fetomaternal outcome.

## INTRODUCTION

Induction of labor is used for several maternal and fetal indications across the world to improve pregnancy outcomes. Induction of labor (IOL) is the technique of initiation of uterine contractions in pregnant women who are not in spontaneous labor to assist them in having a vaginal birth within 24 to 48 hours. Cervical ripening is one way of inducing labor; it is defined as the use of pharmacological or other mechanical methods to soften, efface, and dilate the cervix to improve the possibility of vaginal birth.^1^ Oral misoprostol has been widely considered as a labor inducing agent (Prostaglandin E1).

The World Health Organization (WHO), the International Federation of Gynecology and Obstetrics (FIGO), and the Society of Obstetricians and Gynecologists of Canada all endorse it for this use (SOGC).^2^,^3^ Misoprostol may be especially useful in the resource-limited countries where alternative prostaglandins (Prostaglandin E2) preparations are expensive and need to be kept in refrigerators for temperature maintenance. It is used widely as oral, sublingual and vaginal routes to induce labour for its high efficacy, considerable safety, reasonable price, easy to use and storage at room temperature.^4^-^6^ Various studies conducted for comparing misoprostol to PGE2 gel for cervical ripening for labor induction at term has indicated that the misoprostol group had shorter induction to delivery interval and a lower cesarean delivery rate.^7^-^9^ Recent research has concentrated on low-dose vaginal misoprostol to be effective while minimizing the frequency of uterine hyperstimulation (25 mcg).

According to the current practice, the optimal misoprostol dose for induction of labor is 25 mcg vaginally every four to six hours.^10^ Misoprostol usage for induction of labor is usually safe in women who have full-term healthy fetuses. Various studies conducted in Pakistan comparing the efficacy of 50ug Misoprostol with Prostaglandin has found Misoprostol to be more effective in terms of shorter duration of labour, but has effect of uterine hyper stimulation and fetal heart rate abnormalities.^11^,^12^ We conducted this study to affirm and observe the effective role of low dose vaginal Misoprostol (25ug) for induction of labor (IOL) in our clinical setting at Lady Reading Hospital, Peshawar, KPK. Pakistan.

## METHODS

A cross-sectional study was carried out at the MTI, Lady Reading Hospital (LRH) Department of Obstetrics and Gynecology in Peshawar, KPK. The study lasted from 21^st^ January 2021 to 31^st^ December 2021.

Sample size was calculated via online sample size calculator, by taking total pregnant population undergoing induction of labour in a year as 500, power of test as 80%, confidence level of 95%, margin of error two and proportion of success of 25ug of misoprostol within six hours as 97.6%.^13^ Three hundred and thirty seven pregnant women who were admitted for IOL (Induction of Labor) having singleton pregnancy with cephalic presentation, were included in this study. Non probability convenient sampling method was used. Informed consent was obtained from all the participants. Cases of previous cesarean scar, multiple pregnancies, non-cephalic presentation, and fetal demise in utero were all excluded. Detailed history and clinical examination were noted for all patients. Pre induction Bishop Score was noted. Induction of labor was started with 25 micrograms of misoprostol, repeated every six hours depending on Bishop Score. A maximum of six doses were given. Labor progress was monitored on partogram and fetal monitoring was done with regular CTG (Cardiotocograph). The maternal outcome was noted, in terms of mode of delivery and need for a C-section. The fetal outcome was measured in terms of APGAR score and need for NICU (Neonatal Intensive Care Unit) admission. All data was analyzed in SPSS (v 25). Clinical information regarding age, parity, period of gestation, Bishop Score at IOL and time from IOL to onset of labour was noted on a predesigned proforma.

### Ethical Approval

It was obtained from IRB (Institutional Review Board) of the Hospital, IRB no 46/LRH/MTI dated 20^th^ January 2021.

## RESULTS

The means age of women was 26.24(±2.42) years and mean gestational age was 38.34 (± 2.3) weeks. Forty six point two percent were prim gravida. Bishop’s score was less than six in 311 (92.3% of women). It shows that delivery occurred within 6-8 hours of IOL in 60.8% of women and 27.3% of females delivered after eight hours (Table-I).

**Table-I:** Clinical Information n=337

Categories	Frequency (%)	Percentage
***Age Distribution***		
Less than 18	10	3%
18-35	256	76%
More than 35	71	21%
***Parity of women***		
Nulliparous	156	46.2%
1-4	125	37.1%
More than 4	56	16.61%
***Bishop Score at IOL***		
Less than 6	311	92.3%
More than 6	26	7.7
***Time from IOL to delivery***		
8 hours	113	33.5%
6 hours	92	27.3%
10 hours	34	10.1%
12 hours	18	5.3%
7 hours	8	2.4%
> 12 hours	72	21.6%
***Period of Gestation***		
37-40 weeks	224	66.46%
>40 weeks	113	33.54%

The indications for IOL, with premature rupture of membranes being the most common at 32.9%. It is followed closely by post-dates and eclampsia (30.6% and 27.6%) respectively. Three doses of misoprostol were required in 105(31.2%) females for the induction. Only 19 (5.6%) females required six doses of misoprostol for induction (Table-II & III).

**Table-II:** Indications for IOL

Indications	Frequency	Percent
Eclampsia / Pre-eclampsia	93	27.6
Postdate pregnancy	103	30.6
Premature Rupture of Membranes	111	32.9
Gestational Diabetes	11	3.3
Other Medical Disorders	7	2.1
Bad Obstetrical History	7	2.1
Polyhydramnios	2	0.6
Antepartum hemorrhage	3	0.9

Total	337	100.0

**Table-III:** Number of Misoprostol Doses.

Dose #	Frequency	Percent
Dose 1	37	11.0
Dose 2	81	24.0
Dose 3	105	31.2
Dose 4	80	23.7
Dose 5	15	4.5
Dose 6	19	5.6

Total	337	100.0

Fetal outcome in terms of APGAR score which was 8/10 in 84% of newborn at one minute and 10/10 in 88.1% at five minute. There was no meconium staining of liquor in 80% of women (Table-IV). After successful induction of labor with Misoprostol 287 (85.1%) females delivered spontaneously, 2.37% required forceps delivery, 6 (1.7%) required vacuum delivery, and 36(10.68%) delivered by Caesarean Section (Table-V).

**Table-IV:** Fetal outcome in terms of APGAR.

Categories	Frequency	Percentage
***Neonatal ICU Admission***		
Yes	41	12.1%
No	296	87.8%
***APGAR Score (1 minute)***		
<4	2	0.6%
4/10	8	2.4%
5/10	2	0.6
6/10	42	12.5%
8/10	283	84%
***APGAR Score (5 minutes)***		
4/10	1	0.3%
6/10	4	1.2%
8/10	35	10.4%
10/10	297	88.1%
***Meconium Staining of Liquor***		
No Meconium	270	80.1%
Grade 1	51	15.1%
Grade 2	8	2.4%
Grade 3	8	2.4%

**Table-V:** Maternal Outcome / Mode of Delivery.

Mode of Delivery	Frequency	Percent
Normal Vaginal Delivery	287	85.1
Forceps Delivery	8	2.37
Vacuum Delivery	6	1.7
Emergency Caesarean Section	36	10.68

Total	337	100.0

## DISCUSSION

The use of misoprostol for induction of labour has been increasing worldwide. There is increasing evidence that misoprostol administered either through vaginal or oral route is as effective as other pharmacological methods for IOL at term.^14^,^15^ The majority of the women in the present study were prim gravida, requiring just three doses of 25microgram vaginal misoprostol for induction and the Bishop score was less than six in 92.3% of females. Misoprostol caused 85.1% of females to deliver spontaneously and 10.68 percent to deliver through Caesarean Section. Indications for emergency caesarean section were fetal distress with pathological CTG, meconium stained liquor and failure to progress. One minute APGAR score was 8/10 in 84% of newborns and there was no admission to NICU in 88% of neonates. There was no case of uterine hyper stimulation in women in whom maximum of six doses were given. Main problem reported with vaginal misoprostol is uterine hyper stimulation and excessive contractions which is common with high dose and specially when used in multigravida.^16^

Earlier studies have reported that when misoprostol is administered correctly, the absolute hazards are modest. Vaginal misoprostol reduced the time from IOL to vaginal birth compared to other techniques of labor induction and augmentation, but it did not lower the rate of the cesarean section when compared to oral misoprostol. The advantage of a faster birth with a misoprostol vaginal insertion should be balanced against the increased risks of uterine hyper stimulation and meconium-stained amniotic fluid.^17^ These complications are common in multigravida women, and our study included majority of prim gravidae women so didn’t find any case of uterine hyper stimulation even in women (5.6%) where six doses were used.

Low-dose misoprostol used orally rather than vaginally is likely to result in comparable rates of vaginal delivery; however, rates may be lower within the first 24 hours. According to the best-known research, low-dose vaginal misoprostol offers significant advantages over other techniques of inducing labor. The majority of women (60.5%) in our study delivered within 6-8 hours of IOL. We find no evidence of uterine hyper stimulation in our patients. Earlier study supports the use of low-dose vaginal misoprostol for induction of labor and shows that the risks of hyperstimulation are fewer than 50ug misoprostol.^18^

These studies support the results of our study in terms of short induction to delivery interval, more spontaneous vaginal deliveries and good fetal outcome. Higher misoprostol dosages are frequently associated with uterine contraction irregularities. significantly higher in the 50-microg misoprostol group (26.8 vs. 8.6%; p Some trials also show an increase in in the 25-microg group more women achieved vaginal delivery (79.3 vs. 60.7%; p < 0.05). The rate of cesarean sections due to non-reassuring fetal status was higher in the 50-microg misoprostol group (28.6 vs. 10.3%; p < 0.05). The number of neonates with a low one-minute Apgar score (<7) was < 0.05)^19^meconium staining of liquor, neonatal acidemia, and cesarean delivery for fetal distress.^19^,^20^

When administered wisely and cautiously, misoprostol is an effective medication for cervical softening and labor induction.^21^,^22^ Although a dose of 50 microgram of misoprostol results in a significantly shorter induction-delivery interval with less need for labor augmentation, there was an increased risk of uterine contractile abnormalities and postpartum hemorrhage.^23^,^24^

Previous studies in Pakistan compared the 50µg dose of Misoprostol with Prostaglandin E2, and found the Misoprostol to be more effective in terms of reduced duration of labour but increased risk of uterine hyper stimulation, so low dose Misoprostol can be used with minimal side effects.^25^,^26^ Nisa Q studied 50 µg oral misoprostol for IOL in women with PROM and found labour delivery interval to be shortened in multigravida compared to primigravida.^27^

A regime using 25 microgram of misoprostol every six hours can induce labor safely and effectively. In present study, Misoprostol 25µg used vaginally with maximum women delivering with three doses and maternal and fetal outcome were comparable to other studies. FIGO (2017) recommended 25µg misoprostol vaginally every six hours regimens for induction of labour. Twenty-five 25µg of misoprostol should be considered as the initial dose for cervical ripening and labour induction.^28^

### Limitation

There is need for large scale comparative studies to validate the efficacy and safety of 25 µg vaginal misoprostol vs. 50 µg misoprostol. Majority of women in our study were prim gravida with no risk of uterine hyper stimulation. We need to start use of vaginal misoprostol in multigravida women for IOL.

## CONCLUSION

Misoprostol is cheap and effective drug for the induction of labor. We found Misoprostol to be economical, easy to use and effective in low doses (25µg) in our set up.

### Recommendations

Misoprostol shows promise as a highly effective, low-cost, and simple agent for labor induction, especially in developing countries. Misoprostol regimens with lower doses should be studied further with larger group as participants.

### Authors contribution:

**LZ:** Conceptualization.

**TS:** Study Design and Execution.

**SSF** & **RB:** Data Acquisition and Analysis.

**TS** & **LZ:** Revised and Critically Reviewed.

**LZ:** Responsible for the integrity and accuracy of the study.
